# (1*E*,4*E*)-1-(2-Nitro­phen­yl)-5-(2,6,6-trimethyl­cyclo­hex-1-en-1-yl)penta-1,4-dien-3-one

**DOI:** 10.1107/S1600536812022453

**Published:** 2012-05-23

**Authors:** Peng Zou, Yi-Jun Jin, Liu-Fang Xiang, Dong-Ping Sun, Shu-Lin Yang

**Affiliations:** a Institute of Biotechnology, Nanjing University of Science and Technology, Nanjing, Jiangsu Province 210094, People’s Republic of China

## Abstract

In the title curcumin–ionone derivative, C_20_H_23_NO_3_, the dihedral angle between the cyclo­hexene and benzene rings is 21.03 (8)°, with both double bonds in the inter­linking olefinic chain adopting *E* conformations. Two of the methyl­ene groups of the β-ionone ring are disordered over two sets of sites with occupancy ratios of 0.50:0.50 and 0.60:0.40. In the crystal, mol­ecules are linked by weak C—H⋯O hydrogen bonds into zigzag chains extending along the *b* axis.

## Related literature

For related structures, see: Liang *et al.* (2007 ▶); Zhang *et al.* (2012 ▶). For background to the biological properties of curcumin–ionone derivatives, see: Asokkumar *et al.* (2012 ▶); Hsu & Cheng (2007 ▶); Kuttan *et al.* (1985 ▶); Zhao, Cai *et al.* (2010 ▶); Zhao, Yang *et al.* (2010 ▶).
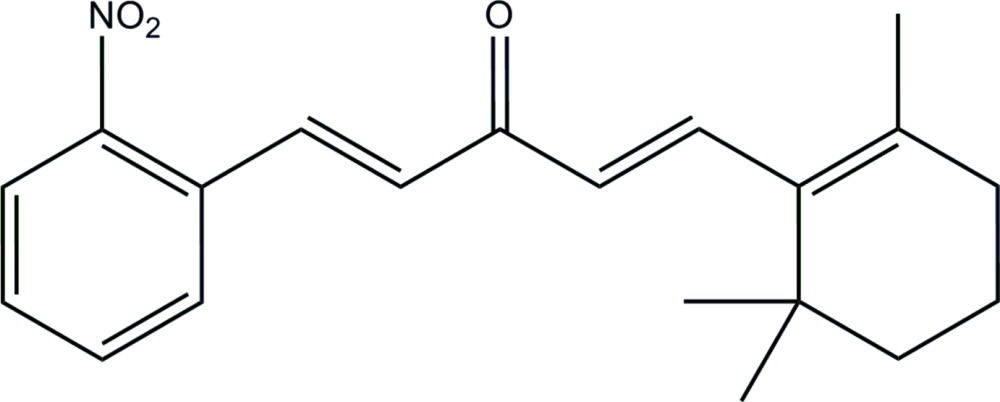


## Experimental

### 

#### Crystal data

C_20_H_23_NO_3_
*M* *_r_* = 325.39
Monoclinic, 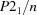

*a* = 7.2941 (6) Å
*b* = 19.2984 (15) Å
*c* = 12.7491 (10) Å
β = 92.892 (2)°
*V* = 1792.3 (2) Å^3^
*Z* = 4
Mo *K*α radiation
μ = 0.08 mm^−1^
*T* = 293 K
0.33 × 0.25 × 0.08 mm

#### Data collection

Bruker SMART CCD area-detector diffractometer
Absorption correction: multi-scan (*SADABS*; Bruker, 2002 ▶) *T*_min_ = 0.533, *T*_max_ = 1.000
10762 measured reflections
3512 independent reflections
2629 reflections with *I* > 2σ(*I*)
*R*_int_ = 0.029

#### Refinement

*R*[*F* ^2^ > 2σ(*F* ^2^)] = 0.055
*wR*(*F* ^2^) = 0.144
*S* = 1.06
3512 reflections
238 parameters
H-atom parameters constrained
Δρ_max_ = 0.23 e Å^−3^
Δρ_min_ = −0.14 e Å^−3^

### 

Data collection: *SMART* (Bruker, 2002 ▶); cell refinement: *SAINT* (Bruker, 2002 ▶); data reduction: *SAINT*; program(s) used to solve structure: *SHELXS97* (Sheldrick, 2008 ▶); program(s) used to refine structure: *SHELXL97* (Sheldrick, 2008 ▶); molecular graphics: *SHELXTL* (Sheldrick, 2008 ▶); software used to prepare material for publication: *SHELXTL*.

## Supplementary Material

Crystal structure: contains datablock(s) I, global. DOI: 10.1107/S1600536812022453/zs2210sup1.cif

Structure factors: contains datablock(s) I. DOI: 10.1107/S1600536812022453/zs2210Isup2.hkl

Supplementary material file. DOI: 10.1107/S1600536812022453/zs2210Isup3.cml

Additional supplementary materials:  crystallographic information; 3D view; checkCIF report

## Figures and Tables

**Table 1:** Hydrogen-bond geometry (Å, °)

*D*—H⋯*A*	*D*—H	H⋯*A*	*D*⋯*A*	*D*—H⋯*A*
C8—H8⋯O1^i^	0.93	2.50	3.182 (2)	131

Symmetry code: (i) 
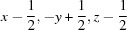
.

**Fig. 1.**
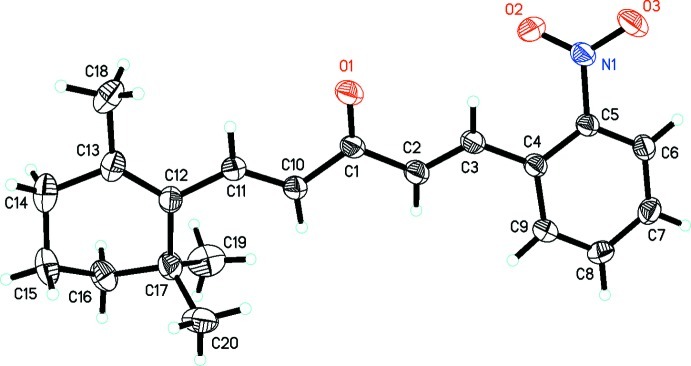
The molecular structure of the title compound, showing 30% displacement ellipsoids for the non-hydrogen atoms. The disordered components of C15 and C16 are not shown. Hydrogen atoms are drawn as spheres of arbitrary radius.

## supplementary crystallographic information

### Comment

Ionone is an important intermediate in the metabolism of terpenoids, and has been isolated from many sources, and represents a promising candidate for chemopreventive applications. Ionone has been used for *in vivo* and *in vitro* protection against various types of cancer cells (Asokkumar *et al.*, 2012). Curcumin is a yellow compound isolated from the rhizome of the herb *Curcuma longa L*, which has been used for centuries as a dietary pigment, spice, and traditional medicine in India and China (Kuttan *et al.*, 1985). Several clinical trials involving curcumin are currently being conducted on patients with pancreatic cancer, multiple myeloma, rheumatoid arthritis, cystic fibrosis, inflammatory bowel disease, psoriasis, and other disorders (Hsu & Cheng, 2007). Our previous studies also showed that some monocarbonyl analogues of curcumin without the β-diketone moiety exhibited better anti-inflammatory activities than those of curcumin (Liang *et al.*, 2007; Zhao, Cai *et al.*, 2010; Zhao, Yang *et al.*, 2010).

In the present study, we designed and synthesized a series of ionone-based monocarbonyl analogues of curcumin by incorporating ionone and monocarbonyl dienone into one chemical entity. One of these was the title compound, C_20_H_23_NO_3_ and its structure is reported here. In the molecule (Fig. 1) the dihedral angle between the cyclohexene ring and the benzene ring is 21.03 (8)° with both double bonds in the inter-linking olefinic chain adopting *E*-configurations. Two of the methylene groups (C15 and C16) of the β-ionone ring are disordered over two sites, C15' (S.O.F = 0.40) and C16' (S.O.F. = 0.50), respectively. In the crystal, the molecules are linked through a weak intermolecular aromatic C—H···O_carbonyl_ hydrogen bond, (Table 1), giving zigzag chains which extend down the *b*-cell direction.

### Experimental

To the mixture of β-ionone (2.5 mmol, 0.481 g) and 2-nitrobenzaldehyde (2.5 mmol) in 10 ml of ethanol, 1 ml of 10% NaOH was added and the mixture was stirred for 12 h at room temperature. After addition of 10 ml of water, the solution was extracted by 3×10 ml of CH_2_Cl_2_. The crude product was obtained from the combined organic layers, and was purified by silica gel column chromatography (elutant: EtOAc/hexane). Crystals of the title compound suitable for X-ray analysis were obtained from an ethanol/chloroform solution (1:3, v/v) at 293 K.

### Refinement

Hydrogen atoms were positioned geometrically, with C—H = 0.93 Å (aromatic or olefinic), 0.96 Å (methyl) or 0.97 Å (methylene) and were allowed to ride on their parent atoms, with *U*_iso_(H) = 1.2*U*_eq_(aromatic, olefinic or methylene C) or 1.5*U*_eq_ (methyl C). The methyl groups C15 and C16 were found to be disordered over two sites (C15' and C16') with occupancies of 0.60/0.40 and 0.50/0.50, respectively.

### Crystal data

**Table:**

C_20_H_23_NO_3_	*F*(000) = 696
*M**_r_* = 325.39	*D*_x_ = 1.206 Mg m^−^^3^
Monoclinic, *P*2_1_/*n*	Mo *K*α radiation, λ = 0.71073 Å
Hall symbol: -P 2yn	Cell parameters from 2030 reflections
*a* = 7.2941 (6) Å	θ = 5.3–42.7°
*b* = 19.2984 (15) Å	µ = 0.08 mm^−^^1^
*c* = 12.7491 (10) Å	*T* = 293 K
β = 92.892 (2)°	Prismatic, green
*V* = 1792.3 (2) Å^3^	0.33 × 0.25 × 0.08 mm
*Z* = 4	

### Data collection

**Table:**

Bruker SMART CCD area-detector diffractometer	3512 independent reflections
Radiation source: fine-focus sealed tube	2629 reflections with *I* > 2σ(*I*)
Graphite monochromator	*R*_int_ = 0.029
φ and ω scans	θ_max_ = 26.0°, θ_min_ = 1.9°
Absorption correction: multi-scan (*SADABS*; Bruker, 2002)	*h* = −8→8
*T*_min_ = 0.533, *T*_max_ = 1.000	*k* = −23→19
10762 measured reflections	*l* = −15→15

### Refinement

**Table:**

Refinement on *F*^2^	Primary atom site location: structure-invariant direct methods
Least-squares matrix: full	Secondary atom site location: difference Fourier map
*R*[*F*^2^ > 2σ(*F*^2^)] = 0.055	Hydrogen site location: inferred from neighbouring sites
*wR*(*F*^2^) = 0.144	H-atom parameters constrained
*S* = 1.06	*w* = 1/[σ^2^(*F*_o_^2^) + (0.0618*P*)^2^ + 0.407*P*] where *P* = (*F*_o_^2^ + 2*F*_c_^2^)/3
3512 reflections	(Δ/σ)_max_ = 0.001
238 parameters	Δρ_max_ = 0.23 e Å^−^^3^
0 restraints	Δρ_min_ = −0.14 e Å^−^^3^

### Special details

**Table:**

Geometry. All e.s.d.'s (except the e.s.d. in the dihedral angle between two l.s. planes) are estimated using the full covariance matrix. The cell e.s.d.'s are taken into account individually in the estimation of e.s.d.'s in distances, angles and torsion angles; correlations between e.s.d.'s in cell parameters are only used when they are defined by crystal symmetry. An approximate (isotropic) treatment of cell e.s.d.'s is used for estimating e.s.d.'s involving l.s. planes.
Refinement. Refinement of F2 against all reflections. The weighted *R*-factor *wR* and goodness of fit S are based on F2, conventional Rfactors *R* are based on F, with F set to zero for negative F2. The threshold expression of F2 > 2sigma(F2) is used only for calculating Rfactors (gt) *etc*. and is not relevant to the choice of reflections for refinement. *R*-factors based on F2 are statistically about twice as large as those based on F, and R– factors based on all data will be even larger.

### Fractional atomic coordinates and isotropic or equivalent isotropic displacement parameters (Å^2^)

**Table:**

	*x*	*y*	*z*	*U*_iso_*/*U*_eq_	Occ. (<1)
N1	0.0393 (2)	0.29601 (10)	0.52846 (12)	0.0552 (5)	
O1	0.67912 (19)	0.20268 (9)	0.48739 (10)	0.0668 (5)	
O2	0.1133 (2)	0.24512 (10)	0.56699 (11)	0.0761 (5)	
O3	−0.0291 (3)	0.34121 (11)	0.57997 (12)	0.0906 (6)	
C1	0.6322 (2)	0.18763 (10)	0.39702 (13)	0.0458 (5)	
C2	0.4555 (3)	0.21296 (11)	0.34870 (14)	0.0486 (5)	
H2	0.4212	0.1990	0.2807	0.058*	
C3	0.3453 (2)	0.25430 (10)	0.39799 (14)	0.0447 (4)	
H3	0.3813	0.2676	0.4661	0.054*	
C4	0.1692 (2)	0.28111 (9)	0.35343 (13)	0.0410 (4)	
C5	0.0265 (2)	0.30303 (9)	0.41389 (13)	0.0413 (4)	
C6	−0.1358 (3)	0.32970 (10)	0.37105 (15)	0.0487 (5)	
H6	−0.2263	0.3451	0.4143	0.058*	
C7	−0.1623 (3)	0.33330 (10)	0.26406 (15)	0.0530 (5)	
H7	−0.2733	0.3493	0.2341	0.064*	
C8	−0.0240 (3)	0.31316 (11)	0.20119 (15)	0.0536 (5)	
H8	−0.0411	0.3161	0.1285	0.064*	
C9	0.1391 (3)	0.28873 (10)	0.24514 (14)	0.0489 (5)	
H9	0.2323	0.2769	0.2014	0.059*	
C10	0.7434 (3)	0.14433 (10)	0.33055 (14)	0.0468 (5)	
H10	0.7035	0.1366	0.2611	0.056*	
C11	0.8992 (3)	0.11580 (10)	0.36674 (14)	0.0465 (5)	
H11	0.9313	0.1265	0.4364	0.056*	
C12	1.0294 (3)	0.07069 (10)	0.31721 (15)	0.0468 (5)	
C13	1.1754 (3)	0.04690 (10)	0.37653 (17)	0.0540 (5)	
C14	1.3181 (3)	0.00004 (14)	0.3345 (2)	0.0806 (8)	
H14A	1.3006	−0.0464	0.3612	0.097*	0.60
H14B	1.4386	0.0158	0.3598	0.097*	0.60
H14C	1.3564	−0.0326	0.3880	0.097*	0.40
H14D	1.4220	0.0272	0.3161	0.097*	0.40
C15	1.3113 (7)	−0.0023 (4)	0.2146 (5)	0.0806 (15)	0.60
H15A	1.3534	0.0414	0.1868	0.097*	0.60
H15B	1.3898	−0.0390	0.1908	0.097*	0.60
C16	1.1135 (8)	−0.0156 (3)	0.1771 (5)	0.0617 (14)	0.50
H16A	1.0659	−0.0555	0.2130	0.074*	0.50
H16B	1.1065	−0.0249	0.1022	0.074*	0.50
C15'	1.2479 (15)	−0.0375 (4)	0.2337 (8)	0.082 (3)	0.40
H15C	1.3461	−0.0635	0.2035	0.099*	0.40
H15D	1.1491	−0.0691	0.2485	0.099*	0.40
C16'	1.1810 (10)	0.0186 (4)	0.1612 (5)	0.0762 (16)	0.50
H16C	1.1588	0.0000	0.0910	0.091*	0.50
H16D	1.2736	0.0546	0.1582	0.091*	0.50
C17	0.9974 (3)	0.05041 (11)	0.20138 (16)	0.0582 (6)	
C18	1.2140 (3)	0.06430 (14)	0.49096 (18)	0.0751 (7)	
H18A	1.1982	0.1132	0.5013	0.113*	
H18B	1.3378	0.0515	0.5115	0.113*	
H18C	1.1305	0.0393	0.5328	0.113*	
C19	0.8125 (4)	0.01320 (13)	0.1825 (2)	0.0835 (8)	
H19A	0.7145	0.0462	0.1855	0.125*	
H19B	0.7994	−0.0214	0.2357	0.125*	
H19C	0.8082	−0.0085	0.1147	0.125*	
C20	1.0017 (3)	0.11384 (14)	0.13065 (17)	0.0729 (7)	
H20A	0.9097	0.1462	0.1504	0.109*	
H20B	0.9777	0.1001	0.0589	0.109*	
H20C	1.1205	0.1352	0.1381	0.109*	

### Atomic displacement parameters (Å^2^)

**Table:**

	*U*^11^	*U*^22^	*U*^33^	*U*^12^	*U*^13^	*U*^23^
N1	0.0414 (9)	0.0854 (13)	0.0391 (9)	0.0153 (9)	0.0032 (7)	−0.0070 (9)
O1	0.0511 (9)	0.1112 (13)	0.0381 (8)	0.0193 (8)	0.0009 (6)	−0.0105 (8)
O2	0.0691 (11)	0.1120 (14)	0.0473 (8)	0.0321 (10)	0.0044 (7)	0.0175 (9)
O3	0.0924 (13)	0.1301 (16)	0.0492 (9)	0.0438 (12)	0.0039 (8)	−0.0284 (10)
C1	0.0395 (10)	0.0623 (12)	0.0361 (10)	0.0027 (9)	0.0070 (8)	0.0016 (8)
C2	0.0431 (10)	0.0630 (12)	0.0396 (10)	0.0048 (9)	0.0018 (8)	−0.0062 (9)
C3	0.0395 (10)	0.0578 (12)	0.0370 (9)	0.0027 (8)	0.0034 (8)	−0.0042 (8)
C4	0.0409 (10)	0.0440 (10)	0.0382 (9)	0.0021 (8)	0.0027 (8)	−0.0044 (7)
C5	0.0402 (10)	0.0475 (10)	0.0359 (9)	0.0036 (8)	0.0009 (7)	−0.0039 (8)
C6	0.0445 (11)	0.0530 (12)	0.0487 (11)	0.0098 (9)	0.0025 (9)	−0.0063 (9)
C7	0.0513 (12)	0.0538 (12)	0.0527 (11)	0.0118 (9)	−0.0086 (9)	0.0015 (9)
C8	0.0625 (13)	0.0603 (12)	0.0372 (10)	0.0070 (10)	−0.0048 (9)	0.0028 (9)
C9	0.0524 (12)	0.0562 (12)	0.0388 (10)	0.0058 (9)	0.0083 (8)	−0.0018 (8)
C10	0.0419 (11)	0.0607 (12)	0.0377 (9)	0.0065 (9)	0.0013 (8)	−0.0019 (8)
C11	0.0439 (11)	0.0551 (11)	0.0405 (9)	0.0036 (9)	0.0021 (8)	0.0018 (8)
C12	0.0426 (10)	0.0472 (10)	0.0507 (11)	0.0051 (8)	0.0030 (8)	0.0026 (8)
C13	0.0448 (11)	0.0490 (11)	0.0679 (13)	0.0027 (9)	−0.0014 (10)	0.0089 (10)
C14	0.0597 (15)	0.0751 (16)	0.106 (2)	0.0257 (13)	−0.0002 (14)	0.0025 (15)
C15	0.059 (3)	0.081 (4)	0.102 (4)	0.028 (3)	0.016 (3)	−0.010 (3)
C16	0.060 (4)	0.051 (3)	0.075 (3)	0.001 (3)	0.015 (3)	−0.012 (3)
C15'	0.086 (6)	0.048 (4)	0.115 (6)	0.025 (4)	0.027 (5)	−0.001 (4)
C16'	0.074 (5)	0.076 (4)	0.079 (4)	0.019 (4)	0.017 (3)	−0.018 (3)
C17	0.0579 (13)	0.0619 (13)	0.0546 (12)	0.0157 (11)	0.0029 (10)	−0.0105 (10)
C18	0.0665 (15)	0.0839 (17)	0.0724 (15)	0.0090 (13)	−0.0204 (12)	0.0140 (13)
C19	0.112 (2)	0.0615 (15)	0.0759 (16)	−0.0227 (15)	−0.0086 (15)	−0.0128 (12)
C20	0.0743 (16)	0.0969 (18)	0.0482 (12)	−0.0140 (14)	0.0103 (11)	0.0054 (12)

### Geometric parameters (Å, º)

**Table:**

N1—O2	1.213 (2)	C14—C15'	1.539 (10)
N1—O3	1.214 (2)	C14—H14A	0.9700
N1—C5	1.465 (2)	C14—H14B	0.9700
O1—C1	1.220 (2)	C14—H14C	0.9600
C1—C10	1.464 (3)	C14—H14D	0.9600
C1—C2	1.483 (3)	C15—C16	1.519 (8)
C2—C3	1.314 (3)	C15—H15A	0.9700
C2—H2	0.9300	C15—H15B	0.9700
C3—C4	1.472 (3)	C16—C17	1.569 (6)
C3—H3	0.9300	C16—H16A	0.9700
C4—C5	1.392 (2)	C16—H16B	0.9700
C4—C9	1.395 (2)	C15'—C16'	1.490 (11)
C5—C6	1.378 (2)	C15'—H15C	0.9700
C6—C7	1.370 (3)	C15'—H15D	0.9700
C6—H6	0.9300	C16'—C17	1.582 (6)
C7—C8	1.376 (3)	C16'—H16C	0.9700
C7—H7	0.9300	C16'—H16D	0.9700
C8—C9	1.372 (3)	C17—C20	1.522 (3)
C8—H8	0.9300	C17—C19	1.536 (3)
C9—H9	0.9300	C18—H18A	0.9600
C10—C11	1.325 (3)	C18—H18B	0.9600
C10—H10	0.9300	C18—H18C	0.9600
C11—C12	1.455 (3)	C19—H19A	0.9600
C11—H11	0.9300	C19—H19B	0.9600
C12—C13	1.355 (3)	C19—H19C	0.9600
C12—C17	1.534 (3)	C20—H20A	0.9600
C13—C14	1.498 (3)	C20—H20B	0.9600
C13—C18	1.510 (3)	C20—H20C	0.9600
C14—C15	1.527 (7)		
			
O2—N1—O3	123.32 (17)	C15'—C14—H14D	106.7
O2—N1—C5	118.91 (16)	H14C—C14—H14D	109.1
O3—N1—C5	117.74 (17)	C16—C15—C14	107.6 (4)
O1—C1—C10	123.06 (17)	C16—C15—H15A	110.2
O1—C1—C2	120.66 (17)	C14—C15—H15A	110.2
C10—C1—C2	116.28 (15)	C16—C15—H15B	110.2
C3—C2—C1	122.75 (17)	C14—C15—H15B	110.2
C3—C2—H2	118.6	H15A—C15—H15B	108.5
C1—C2—H2	118.6	C15—C16—C17	108.3 (4)
C2—C3—C4	124.85 (17)	C15—C16—H16A	110.0
C2—C3—H3	117.6	C17—C16—H16A	110.0
C4—C3—H3	117.6	C15—C16—H16B	110.0
C5—C4—C9	115.53 (16)	C17—C16—H16B	110.0
C5—C4—C3	123.75 (15)	H16A—C16—H16B	108.4
C9—C4—C3	120.67 (16)	C16'—C15'—C14	105.1 (6)
C6—C5—C4	123.06 (16)	C16'—C15'—H15C	110.7
C6—C5—N1	116.09 (16)	C14—C15'—H15C	110.7
C4—C5—N1	120.80 (15)	C16'—C15'—H15D	110.7
C7—C6—C5	119.28 (18)	C14—C15'—H15D	110.7
C7—C6—H6	120.4	H15C—C15'—H15D	108.8
C5—C6—H6	120.4	C15'—C16'—C17	109.7 (6)
C6—C7—C8	119.66 (18)	C15'—C16'—H16C	109.7
C6—C7—H7	120.2	C17—C16'—H16C	109.7
C8—C7—H7	120.2	C15'—C16'—H16D	109.7
C9—C8—C7	120.33 (17)	C17—C16'—H16D	109.7
C9—C8—H8	119.8	H16C—C16'—H16D	108.2
C7—C8—H8	119.8	C20—C17—C12	111.00 (18)
C8—C9—C4	122.04 (18)	C20—C17—C19	109.12 (19)
C8—C9—H9	119.0	C12—C17—C19	111.07 (19)
C4—C9—H9	119.0	C20—C17—C16	120.6 (3)
C11—C10—C1	121.74 (17)	C12—C17—C16	109.9 (3)
C11—C10—H10	119.1	C19—C17—C16	93.9 (3)
C1—C10—H10	119.1	C20—C17—C16'	94.4 (3)
C10—C11—C12	131.64 (18)	C12—C17—C16'	108.7 (3)
C10—C11—H11	114.2	C19—C17—C16'	121.3 (3)
C12—C11—H11	114.2	C13—C18—H18A	109.5
C13—C12—C11	118.18 (18)	C13—C18—H18B	109.5
C13—C12—C17	121.84 (18)	H18A—C18—H18B	109.5
C11—C12—C17	119.97 (16)	C13—C18—H18C	109.5
C12—C13—C14	123.1 (2)	H18A—C18—H18C	109.5
C12—C13—C18	124.52 (19)	H18B—C18—H18C	109.5
C14—C13—C18	112.42 (18)	C17—C19—H19A	109.5
C13—C14—C15	112.8 (3)	C17—C19—H19B	109.5
C13—C14—C15'	112.0 (4)	H19A—C19—H19B	109.5
C13—C14—H14A	109.0	C17—C19—H19C	109.5
C15—C14—H14A	109.0	H19A—C19—H19C	109.5
C13—C14—H14B	109.0	H19B—C19—H19C	109.5
C15—C14—H14B	109.0	C17—C20—H20A	109.5
C15'—C14—H14B	133.1	C17—C20—H20B	109.5
H14A—C14—H14B	107.8	H20A—C20—H20B	109.5
C13—C14—H14C	108.9	C17—C20—H20C	109.5
C15'—C14—H14C	110.9	H20A—C20—H20C	109.5
C13—C14—H14D	109.2	H20B—C20—H20C	109.5
			
O1—C1—C2—C3	2.7 (3)	C17—C12—C13—C18	−179.4 (2)
C10—C1—C2—C3	−177.46 (19)	C12—C13—C14—C15	15.3 (4)
C1—C2—C3—C4	179.70 (17)	C18—C13—C14—C15	−164.4 (3)
C2—C3—C4—C5	156.1 (2)	C12—C13—C14—C15'	−20.1 (5)
C2—C3—C4—C9	−26.6 (3)	C18—C13—C14—C15'	160.1 (4)
C9—C4—C5—C6	0.9 (3)	C13—C14—C15—C16	−49.6 (6)
C3—C4—C5—C6	178.25 (18)	C15'—C14—C15—C16	46.0 (7)
C9—C4—C5—N1	178.32 (17)	C14—C15—C16—C17	68.9 (7)
C3—C4—C5—N1	−4.3 (3)	C13—C14—C15'—C16'	54.0 (8)
O2—N1—C5—C6	140.47 (19)	C15—C14—C15'—C16'	−44.3 (6)
O3—N1—C5—C6	−37.9 (3)	C14—C15'—C16'—C17	−71.5 (9)
O2—N1—C5—C4	−37.1 (3)	C13—C12—C17—C20	−118.2 (2)
O3—N1—C5—C4	144.5 (2)	C11—C12—C17—C20	62.8 (2)
C4—C5—C6—C7	2.0 (3)	C13—C12—C17—C19	120.2 (2)
N1—C5—C6—C7	−175.55 (18)	C11—C12—C17—C19	−58.8 (3)
C5—C6—C7—C8	−2.8 (3)	C13—C12—C17—C16	17.7 (4)
C6—C7—C8—C9	0.8 (3)	C11—C12—C17—C16	−161.3 (3)
C7—C8—C9—C4	2.2 (3)	C13—C12—C17—C16'	−15.7 (4)
C5—C4—C9—C8	−3.0 (3)	C11—C12—C17—C16'	165.3 (3)
C3—C4—C9—C8	179.57 (19)	C15—C16—C17—C20	78.9 (5)
O1—C1—C10—C11	3.4 (3)	C15—C16—C17—C12	−52.1 (6)
C2—C1—C10—C11	−176.50 (18)	C15—C16—C17—C19	−166.1 (5)
C1—C10—C11—C12	178.81 (19)	C15—C16—C17—C16'	41.5 (6)
C10—C11—C12—C13	−177.1 (2)	C15'—C16'—C17—C20	165.9 (7)
C10—C11—C12—C17	1.9 (3)	C15'—C16'—C17—C12	52.0 (7)
C11—C12—C13—C14	179.8 (2)	C15'—C16'—C17—C19	−78.5 (7)
C17—C12—C13—C14	0.8 (3)	C15'—C16'—C17—C16	−45.7 (7)
C11—C12—C13—C18	−0.4 (3)		

### Hydrogen-bond geometry (Å, º)

**Table:**

*D*—H···*A*	*D*—H	H···*A*	*D*···*A*	*D*—H···*A*
C8—H8···O1^i^	0.93	2.50	3.182 (2)	131
C9—H9···O1^i^	0.93	2.76	3.318 (2)	119

Symmetry code: (i) *x*−1/2, −*y*+1/2, *z*−1/2.
